# Knockdown of a *nutrient amino acid transporter* gene *LdNAT1* reduces free neutral amino acid contents and impairs *Leptinotarsa decemlineata* pupation

**DOI:** 10.1038/srep18124

**Published:** 2015-12-11

**Authors:** Kai-Yun Fu, Wen-Chao Guo, Tursun Ahmat, Guo-Qing Li

**Affiliations:** 1 Education Ministry Key Laboratory of Integrated Management of Crop Diseases and Pests, College of Plant Protection, Nanjing Agricultural University, Nanjing 210095, China; 2 Department of Plant Protection, Xinjiang Academy of Agricultural Sciences; Urumqi 830091, China

## Abstract

A *Leptinotarsa decemlineata* SLC6 NAT gene (*LdNAT1*) was cloned. *LdNAT1* was highly expressed in the larval alimentary canal especially midgut. *LdNAT1* mRNA levels were high right after the molt and low just before the molt. JH and a JH analog pyriproxyfen activated *LdNAT1* expression. RNAi of an allatostatin gene *LdAS-C* increased JH and upregulated *LdNAT1* transcription. Conversely, silencing of a JH biosynthesis gene *LdJHAMT* decreased JH and reduced *LdNAT1* expression. Moreover, 20E and an ecdysteroid agonist halofenozide repressed *LdNAT1* expression, whereas a decrease in 20E by RNAi of an ecdysteroidogenesis gene *LdSHD* and disruption of 20E signaling by knockdown of *LdE75* and *LdFTZ-F1* activated *LdNAT1* expression. Thus, *LdNAT1* responded to both 20E and JH. Moreover, knockdown of *LdNAT1* reduced the contents of cysteine, histidine, isoleucine, leucine, methionine, phenylalanine and serine in the larval bodies and increased the contents of these amino acids in the larval feces. Furthermore, RNAi of *LdNAT1* inhibited insulin/target of rapamycin pathway, lowered 20E and JH titers, reduced 20E and JH signaling, retarded larval growth and impaired pupation. These data showed that *LdNAT1* was involved in the absorption of several neutral amino acids critical for larval growth and metamorphosis.

Insects usually lack the capacity for *de novo* biosynthesis of ten amino acids (aliphatic: leucine, isoleucine, valine and threonine; aromatic: phenylalanine, tryptophan and histidine; sulfur containing: methionine; basic: arginine and lysine), and must absorb them from food^1^. Insect midgut cells actively absorb these essential amino acids by transporters^2^ that mainly belong to the SoLute Carrier (SLC) superfamily^3^. In total, 9 out of 10 SLC families participate in amino acid transport in insects^1^. Among the 9 SLC families, SLC6 transporters act in the apical membrane of the alimentary canal, and mediate Na^+^/K^+^ ion motive force-coupled transport of amino acids against large chemical gradients^4^. In contrast, the contributions of SLC7, SLC15, SLC36, SLC38 and SLC43 members, to the essential amino acid absorption in midgut, may be secondary or dispensable^1^.

SLC6 family comprises two clearly identifiable subfamilies. The basal subfamily consists of Nutrient Amino acid Transporters (NATs) that mainly absorbs large neutral essential amino acids and proline, known as the B0 (“B” and “0” define the Na^+^-coupled broad substrate spectra for neutral amino acids) and IMINO transporters respectively. The more evolutionary subfamily is the animal-specific neurotransmitter transporters which combine several orthologous clusters of catecholamine (dopamine, norepinephrine and octopamine), indolamine (serotonin) and GABA porters^1^.

In insects, several SLC6 NATs have been cloned from Lepidopteran species *Manduca sexta*^5^,^6^,^7^,^8^,^9^,^10^, and Dipteran species *Aedes aegypti*^11^, *Anopheles gambiae*^12^ and *Drosophila melanogaster*^13^. When expressed in *Xenopus* oocytes, these NATs can absorb a subset of neutral amino acids^14^. However, *in vivo* data have not yet been reported.

A high concentration of free amino acids in insects triggers the insulin/target of rapamycin (TOR) signaling pathways^15^,^16^. Subsequently, insulin/TOR signaling modifies the titers of 20-hydroxyecdysone (20E) and juvenile hormone (JH) to tune larval growth and metamorphosis^17^,^18^,^19^,^20^,^21^,^22^,^23^,^24^. In *Leptinotarsa decemlineata* (Say), a notorious defoliator of potato, several cytochrome P450 monooxygenases such as Spook, Phantom, Disembodied, Shadow, and Shade (SHD) have been documented to be involved in the biosynthesis of ecdysone in the prothoracic gland, and in the production of 20E in the peripheral tissues^25^,^26^. JH has been reported to be produced in the corpora allata. JH acid methyltransferase (JHAMT) participates in JH biosynthesis^27^,^28^. During molting of *L. decemlineata* larvae, either JH or 20E initiates a specific gene expression cascade. Among the activated genes, *Krüppel homolog 1 (LdKr-h1)* is a JH early-inducible gene^28^,^29^, whereas *LdE75* and Ld*FTZ-F1* are 20E-inducible^29^,^30^. During mid instar stage, *L. decemlineata* larvae gnaw a large quantities of potato foliage. At the end of each instar, the larvae stop feeding, and shed their cuticle to allow for further growth. For economical reasons, it appears plausible that, during the molting periods when protein digestion and amino acid absorption are shut down, transcription of relative genes should be downregulated concomitantly, regulated probably by 20E/JH.

In the present paper, we identified a putative *NAT1* gene (*LdNAT1*) in *L. decemlineata*. For the first time in insect species, we tested the induction of *NAT1* by 20E and JH, we knocked down *LdNAT1* by RNA interference (RNAi) to investigate the *in vivo* contributions of *Ld*NAT1 to amino acid absorption in the midgut, as well as to overall beetle biology.

## Methods and Materials

### Insects rearing and chemicals

*L. decemlineata* larvae and adults were routinely reared in an insectary according to a previously described method^28^, and were supplied with potato foliage at vegetative growth or young tuber stages in order to assure sufficient nutrition. At this feeding protocol, *L. decemlineata* larvae progressed through four distinct instars, with the approximate periods of the first-, second-, third- and fourth-instar stages being 2, 2, 2 and 4 days, respectively. Upon reaching full size, the fourth larval instars spent an additional 4–7 days as non-feeding prepupae. The prepupae then dropped to the soil and burrowed to a depth of 3–5 cm to pupate.

An ecdysteroid agonist halofenozide (Hal) (ChemService, West Chester, USA), 20-hydroxyecdysone (20E) (Sigma-Aldrich, USA), juvenile hormone (JH) (Sigma-Aldrich, USA) and a JH analog pyriproxyfen (Pyr) (Ivy Fine Chemicals Corporation, USA) were purified by reverse phase high performance liquid chromatography before experiments.

### Molecular cloning and phylogenetic analysis

Expressed sequence tags of putative *LdNAT1*, *LdInR*, *Ld4EBP*, *LdFOXO*, *LdTOR* and *LdE75* were obtained from *L. decemlineata* transcriptome^31^ and genome data (https://www.hgsc.bcm.edu/arthropods/colorado-potato-beetle-genome-project). The correctness of the sequences was substantiated by polymerase chain reaction (PCR) using primers in Table S1. This was followed by 5′- and 3′-RACE to complete the sequence, with SMARTer RACE cDNA amplification kit (Takara Bio., Dalian, China) and SMARTer RACE kit (Takara Bio.). The antisense/sense gene-specific primers corresponding to the 5′-end and 3′-end of the sequences were listed in Table S1. After obtaining the full-length cDNA, six primer pairs (Table S1) were designed to verify the complete open reading frames. The resulting sequences *LdNAT1*, *LdInR*, *Ld4EBP*, *LdFOXO*, *LdTOR*, *LdE75A*, *LdE75B and LdE75C* were submitted to GenBank with the accession number of AHH29249, KP331063, KP331062, KR075829, KR075825, KP340510, KP340511 and KT246474 respectively. Transmembrane domains of *Ld*NAT1 were predicted using TMHMM 2.0 (www.cbs.dtu.dk/services/TMHMM).

The representative NAT sequences were retrieved from NCBI, and were aligned with the predicted *Ld*NAT1 using ClustalX (2.1)^32^. The neighbor-joining (NJ) tree was constructed using MEGA6^33^ under the Poisson correction method. The reliability of NJ tree topology was evaluated by bootstrapping a sample of 1000 replicates.

### Preparation of dsRNA

The same method as previously described^28^ was used to express ds*SHD*, ds*E75*, ds*FTZ-F1*, ds*JHAMT*, ds*AS-C*, ds*NAT1-*1, ds*NAT1-*2 and ds*egfp* derived from a 141 bp fragment of *LdSHD*, a 361 bp common fragment in the three *LdE75* isoforms, a 319 bp common fragment in both *LdFTZ-F1-1* and *LdFTZ-F1-2* cDNAs, a 261 bp fragment of *LdJHAMT*, a 206 bp fragment of *LdAS-C*, a 665 bp and a 357 bp fragments of *LdNAT1,* and a 414 bp fragment of *enhanced green fluorescent protein* gene. These dsRNAs were individually expressed with specific primers in Table S1, using *Escherichia coli* HT115 (DE3) competent cells lacking RNase III. Individual colonies were inoculated, and induced to express dsRNA by addition of 0.1 mM isopropyl β-D-1-thiogalactopyranoside. The expressed dsRNA was extracted and confirmed by electrophoresis on 1% agarose gel (data not shown), and quantified using a spectrophotometer (NanoDrop Technologies, Wilmington, DE). Bacteria cells were centrifuged at 5000 ×g for 10 min, and resuspended in 0.05 M phosphate buffered saline (PBS, pH 7.4) at the ratio of 1:1. The bacterial suspensions (at dsRNA concentration of about 0.5 μg/ml) were used for bioassay.

### Bioassay

Our preliminary results revealed that feeding the second-instar larvae with 0.1 μg/mL of 20E-, Hal-, JH- or Pyr-immersed foliage did not affect larvae growth, pupation and adult emergence. In this survey, nine independent bioassays were carried out as previously described^25^,^29^, using newly-ecdysed second- and third-instar larvae. The first bioassay was planned to test the effects of 20E and Hal on *LdNAT1* expression. For 1 day, ten third-instar larvae were fed leaves which have been immersed in: (1) water (control), (2) 0.1 μg/mL 20E, or (3) 0.1 μg/mL Hal. Each treatment was replicated three times, and was collected to extract total RNA. The second to fourth bioassays were to knock down *LdSHD*, *LdE75* and *LdFTZ-F1* and each bioassay had three treatments: (1) PBS-, (2) ds*egfp*-, (3) ds*SHD*-, ds*E75*- or ds*FTZ-F1*-immerged leaves. Each treatment (ten larvae) was replicated six times and was fed for 3 days. Three replicates were collected to extract total RNA and the other three replicates were used to extract 20E. The fifth bioassay was intended to determine the effects of JH and Pyr on *LdNAT1* expression by confining ten third-instar larvae for 1 day in petri dishes with: (1) water (control)-, (2) 0.1 μg/mL JH-, or (3) 0.1 μg/mL Pyr-immersed leaves. Three replicates in each treatment were collected to extract total RNA. The sixth and seventh bioassays were intended to silence *LdAS-C* and *LdJHAMT* and had three treatments: (1) PBS-, (2) ds*egfp*-, (3) ds*AS-C*- or ds*JHAMT*-immerged leaves. Six replicates in each treatment (ten larvae) were fed for 3 days to extract total RNA and JH respectively. The eighth and ninth bioassays were planned to knock down *LdNAT1* by allowing ten second- and third-instar larvae to ingest: (1) PBS-, (2) ds*egfp*-, (3) ds*NAT1*-1-, (4) ds*NAT1-*2-dipped leaves. Each treatment was repeated 15 times. For extraction of total RNA, free amino acid, 20E and JH, twelve replicates were respectively collected after continuously fed for 3 days. The remaining 3 replicates were used to measure the larval weight, and to observe the larval developing period and the pupation using the methods as described previously^28^. The surviving larvae were individually weighed 3, 4, 5 and 6 days after treatment. Their development was observed at 4-hr intervals. Larval instars were identified by head capsule width, the appearance of exuviae, the black color of the pronotum, and the anterior beige and posterior black stripe visible on the pronotum of the 3^rd^ and 4^th^ instars respectively. Prepupae were distinctive from larvae by their disappeared black pigmentation, their relative inactivity and their curved body shape. The initiation of pupation was indicated by the soil-digging behavior. The pupation and the adult emergence were recorded during a 4-week trial period. For each bioassay, three biological replicates were carried out.

### Real-time quantitative PCR (qPCR)

For tissue expression analysis, RNA samples were extracted from epidermis, foregut, midgut, hindgut, Malpighian tubules, fat body, hemocytes and brain-corpora cardiaca-corpora allata complex of the day 1 fourth-instar larvae. For temporal expression analysis, RNA templates were derived from the first, second, third larval instars at the interval of one day, and from fourth larval instars at the interval of eight hours. For analysis the effects of bioassay, total RNA was extracted from treated larvae. Each sample contained 5–30 individuals and repeated 3 times. The RNA was extracted using SV Total RNA Isolation System Kit (Promega). Purified RNA was subjected to DNase I to remove any residual DNA according to the manufacturer’s instructions. Quantitative mRNA measurements were performed by qRT-PCR, using internal control genes (*LdRP4*, *LdRP18*, *LdARF1* and *LdARF4*, the primers listed in Table S1) according to our published results^31^. An RT negative control (without reverse transcriptase) and a non-template negative control were included for each primer set to confirm the absence of genomic DNA and to check for primer-dimer or contamination in the reactions, respectively. Each sample was repeated three times. Data were analyzed by the 2^−ΔΔCT^ method, using the geometric mean of internal control genes for normalization. All methods and data were confirmed to follow the MIQE (Minimum Information for publication of Quantitative real time PCR Experiments) guidelines^34^.

### Free amino acid analysis

The larvae were collected from 3 replicates, whereas the feces were collected from all 15 replicates after continuously ingested ds*NAT1*-1 and ds*NAT1*-2 for 3 days. The foliage samples repeated three times. The collected samples were immediately ground in liquid nitrogen and stored at −80 °C until assayed. Hydrolytic amino acids in the foliage were extracted by acid hydrolysis method. Free amino acids in the bodies and feces were extracted with 80% (v/v) ethanol at 25 °C for 10 min. Samples were centrifuged for 15 min at 10000 ×g and 4 °C. Free amino acid contents in the bodies, feces and foliage were analyzed with a Beckman 6300 Amino Acid Analyzer (Beckman Instruments Inc., Fullerton, CA, USA). The amino acid contents were given as μm per gram.

### Quantitative determination of JH and 20E

Hemolymph was collected and JH was extracted following the methods described previously^28^. A liquid chromatography tandem mass spectrometry was used to quantify JH titers (ng per ml hemolymph)^35^.

20E was extracted according to an ultrasonic-assisted extraction method^29^, and its titer (ng per g body weight) was analyzed by a liquid chromatography tandem mass spectrometry-mass spectrometry system using a protocol the same as described^36^.

### Data analysis

The data were given as means ± SE, and were analyzed by analyses of variance (ANOVAs) followed by the Tukey-Kramer test, using SPSS for Windows (SPSS, Chicago, IL, USA). Pupation rate and amino acid composition were subjected to arc-sine transformation before ANOVAs.

## Results

### Identification of a putative nutrient amino acid transporter transcript

We sequenced a full-length transcript encoding a putative nutrient amino acid transporter in *Leptinotarsa decemlineata*, and provisionally designated *LdNAT1*. The correctness of the cDNA was confirmed by end-to-end amplification and sequencing. It was submitted to GenBank with the accession number of AHH29249.

*LdNAT1* had a 1920 bp open reading frame encoding a 640-amino acid protein. The *Ld*NAT1 protein was predicted to have 12 transmembrane domains and intracellular C and N termini using TMHMM 2.0 (Figure S1A), which was in agreement with the general structure of transporters in the SLC6 family^11^. Based on the sequence alignment with selected characterized NATs and a crystallized bacterial NAT^13^,^37^, the possible substrate-binding moieties and first and second sodium-binding sites of *Ld*NAT1 were also predicted (Figure S1A).

The evolutionary relationship of NAT-like representatives derived from 6 insect species was evaluated (Figure S1B). As expected, *LdNAT1* belongs to the Coleopteran clade. It was first grouped with that from *Tribolium castaneum* (XP_973741), with 100% of bootstrap value, and then the two and *T. castaneum* XP_008196787 joined together, supported by 97% of bootstrap value (Figure S1B).

### The expression of LdNAT1

**Figure 1:**
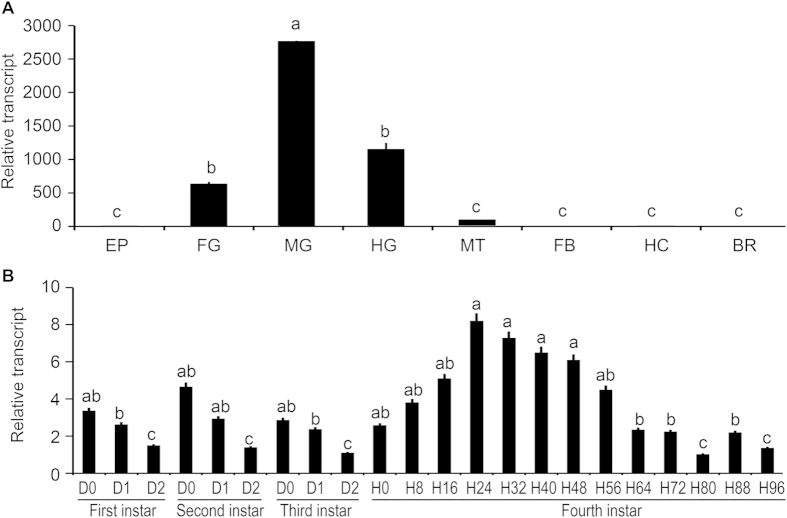
**Tissue (A) and temporal (B) expression patterns of** ***LdNAT1***. For tissue expression analysis, cDNA templates are derived from epidermis (EP), foregut (FG), midgut (MG), hindgut (HG), Malpighian tubules (MT), fat body (FB), hemocytes (HE) and brain-corpora cardiaca-corpora allata complex (BR) of the day 1 fourth-instar larvae. For temporal expression analysis, cDNA templates are derived from the first, second, third larval instars at the interval of one day, and from fourth larval instars at the interval of eight hours (D0/H0 indicated newly ecdysed larvae). For each sample, 3 independent pools of 5–30 individuals are measured in technical triplicate using qPCR. The bars represent 2^-ΔΔCt^ method (±SE) normalized to the geometrical mean of housekeeping gene expression. Different letters indicate significant difference at P value < 0.05.

The tissue expression patterns of *LdNAT1* were tested by quantitative real-time PCR (qRT-PCR). *LdNAT1* was detectable in the epidermis, foregut, midgut, hindgut, Malpighian tubules, fat body, hemocytes and brain-corpora cardiaca-corpora allata complex of the day 1 fourth-instar larvae. *LdNAT1* mRNA was high in the midgut and moderate in the foregut and hindgut, whereas it was expressed at low levels in the Malpighian tubules, epidermis, fat body, hemocytes and brain-corpora cardiaca-corpora allata complex (Fig. 1A).

The temporal expression profiles of *LdNAT1* were also determined in the larvae. *LdNAT1* was expressed throughout all larval stages. Within the first, second and third larval instars, the expression levels of *LdNAT1* were high right after the molt than just before the molt. In the fourth larval instar, a peak of *LdNAT1* occurred 24 hours after ecdysis. Moreover, *LdNAT1* showed two troughs 80 and 96 hours after ecdysis (Fig. 1B).

### Juvenile hormone activates the expression of LdNAT1

**Figure 2:**
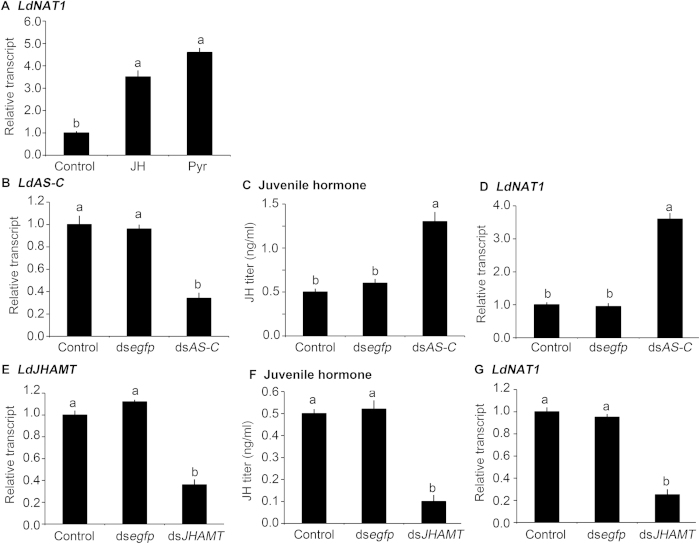
Induction of *LdNAT1* expression by juvenile hormone (JH) in *Leptinotarsa decemlineata*. The newly-ecdysed third-instar larvae have ingested potato leaves immersed with water (control), 0.1 μg/mL Pyr or JH for 1 day (**A**). Otherwise, the newly-ecdysed third-instar larvae have ingested PBS-, ds*egfp*-, or ds*AS-C*-dipped leaves (**B–D**); or PBS-, ds*egfp*-, or ds*JHAMT*-dipped leaves (**E–G**) for 3 days. Different letters indicate significant difference at P value < 0.05.

The expression patterns showed that *LdNAT1* mRNA levels were correlated with circulating JH. To determine whether JH induces *LdNAT1 in vivo*, *LdNAT1* mRNA level in newly-ecdysed *L. decemlineata* third-instar larvae were tested after ingestion of water (control)-, JH- and Pyr-immersed foliage for 1 day. Compared with control specimens, *LdNAT1* expression levels were significantly increased by 3.4 and 4.0 folds in the larvae that had ingested JH and Pyr (Fig. 2A).

Moreover, *LdNAT1* transcription was significantly enhanced by 3.6 fold in the *LdAS-C* RNAi hypomorphs, in which JH titer was significantly increased (Fig. 2B–D).

Furthermore, JHAMT plays a major role in JH biosynthesis^27^,^28^. In this study, we knocked down *LdJHAMT* by RNAi (Fig. 2E) to lower JH titer (Fig. 2F), and found that *LdNAT1* mRNA level was significantly diminished by 61.1% in the *LdJHAMT* RNAi larvae (Fig. 2G).

### 20-Hydroxyecdysone inhibits the expression of LdNAT1

**Figure 3:**
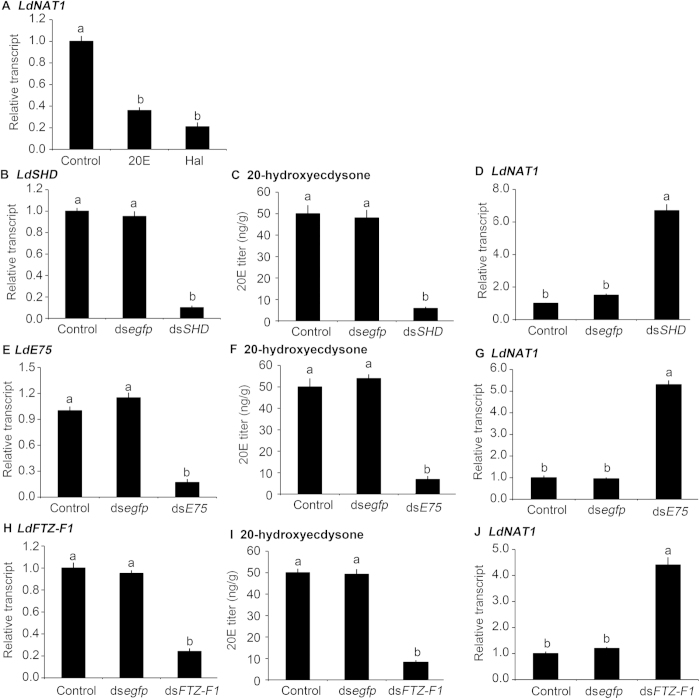
Inhibition of *LdNAT1* expression by 20-hydroxyecdysone (20E) signaling in *Leptinotarsa decemlineata*. The newly-ecdysed third-instar larvae have ingested potato foliage treated with water (control), 0.1 μg/mL halofenozide or 20E for 1 day (**A**). Otherwise, the newly-ecdysed third-instar larvae have ingested PBS-, ds*egfp*-, or ds*SHD*-dipped leaves (**B–D**); or PBS-, ds*egfp*-, or ds*E75*-dipped leaves for 3 days (**E–G**); or PBS-, ds*egfp*-, or ds*FTZ-F1*-dipped leaves (**H–J**) for 3 days. Different letters indicate significant difference at P value < 0.05.

The expression patterns suggest that 20E pulse at the end of each instar inhibits *LdNAT1*. To verify the suggestion, *LdNAT1* mRNA levels in *L. decemlineata* third-instar larvae were tested after ingestion of water (control)-, Hal- and 20E-immersed foliage for 1 day. Compared with control specimens, *LdNAT1* expression levels were significantly decreased by 68.4% and 78.8% in the larvae that had ingested Hal and 20E (Fig. 3A).

Since RNAi of *LdSHD* reduced 20E titer in *L. decemlineata*^25^,^29^, *LdNAT1* mRNA level was tested in the third-instar larvae in which *LdSHD* had been silenced by RNAi (Fig. 3B). As expected, 20E titer had been reduced (Fig. 3C), whereas *LdNAT1* mRNA level was significantly increased by 6.1 fold in the *LdSHD* RNAi hypomorphs, compared with specimens that had ingested ds*egfp* (Fig. 3D).

*Ld*E75 and *Ld*FTZ-F1 mediated 20E signaling in *L. decemlineata*^29^,^30^. In this study, we found three *LdE75* isoforms. We knocked down all these isoforms by dietary introduction of a dsRNA derived from a common fragment in the three *LdE75* isoforms (Fig. 3E). 20E titer was significantly decreased (Fig. 3F), whereas *LdNAT1* transcription was significantly enhanced in the resulting larvae (Fig. 3G). Moreover, knockdown of *LdFTZ-F1* by RNAi (Fig. 3H) also lowered 20E titer (Fig. 3I) but enhanced *LdNAT1* transcription by 3.2 fold (Fig. 3J).

### Knockdown of LdNAT1 affects the absorption of several neutral amino acids

Hydrolytic amino acids in potato foliage, and free amino acids in bodies and feces of the day 1 third- and fourth-instar larvae were analyzed (Tables S2–S4). The compositions of nineteen proteinogenic amino acids (tryptophan is undetectable) were listed in Table 1. The compositions of cysteine, histidine, lysine, methionine, serine, tyrosine and valine in the feces of the third- and fourth-instar larvae were significantly lower than those in the body and the potato foliage. Similarly, the compositions of glutamate, isoleucine, leucine and phenylalanine in the feces of the fourth-instar larvae were significantly lower than those in the body and the potato foliage (Table 1). These data suggest that a total of 11 amino acids (cysteine, histidine, lysine, methionine, serine, tyrosine, valine, glutamate, isoleucine, leucine, phenylalanine) may be actively absorbed by the larval gut.

**Figure 4:**
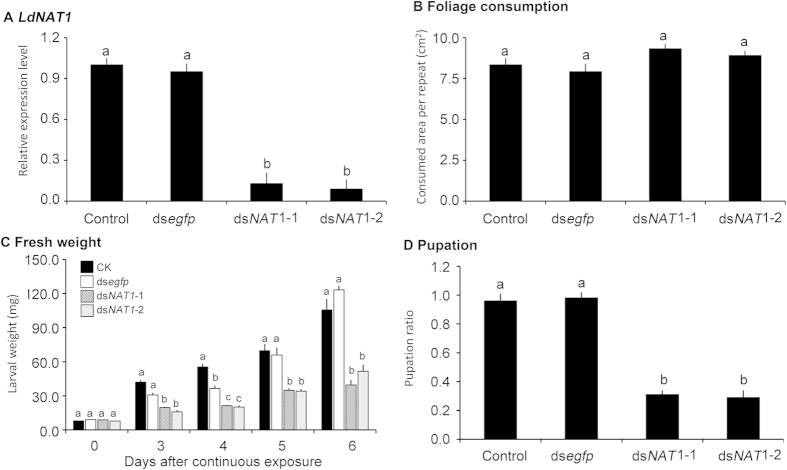
Effects of dietary ingestion of ds*NAT1* (ds*NAT1-*1 and ds*NAT1-*2) by the second-instar *L. decemlineata* larvae on the expression of the target mRNA (A), foliage consumption (B), larval weight (C) and pupation rate (D). For relative transcript determination, 3 independent pools of 5–30 individuals are measured in technical triplicate using qRT-PCR. The bars represent 2^−ΔΔCt^ values (±SE), normalized to the geometrical mean of housekeeping gene expression. For larval weight, larval duration and pupation rate, the bars mean averages (±SE). Different letters indicate significant difference at P value < 0.05.

**Figure 5:**
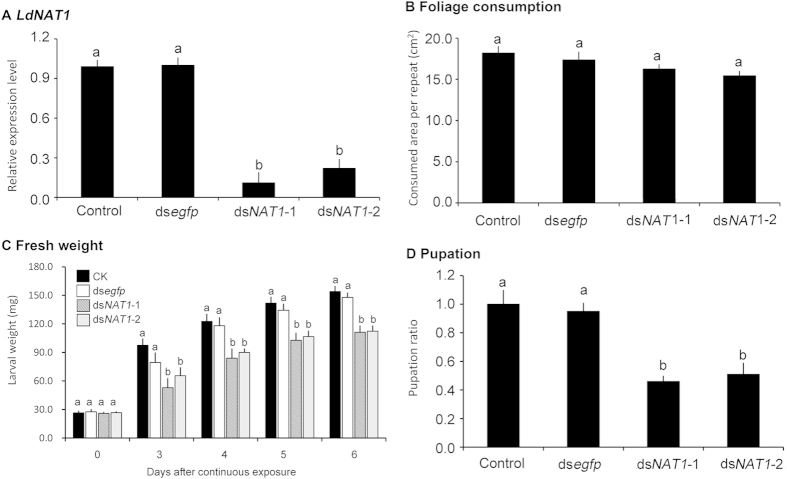
Effects of dietary ingestion of ds*NAT1* (ds*NAT1-*1 and ds*NAT1-*2) by the third-instar *L. decemlineata* larvae on the expression of the target mRNA (A), foliage consumption (B), larval weight (C) and pupation rate (D). For relative transcript determination, 3 independent pools of 5–30 individuals are measured in technical triplicate using qRT-PCR. The bars represent 2^−ΔΔCt^ values (±SE), normalized to the geometrical mean of housekeeping gene expression. For larval weight, larval duration and pupation rate, the bars mean averages (±SE). Different letters indicate significant difference at P value < 0.05.

After the day 1 second- and third-instar larvae had ingested foliage treated with PBS-, ds*egfp*-, ds*NAT1-*1-, ds*NAT1-*2-dipped leaves for three days, the larvae reached to the day 1 of third- and fourth-instar stage respectively. The expression levels of *LdNAT1* in ds*NAT1-*1- and ds*NAT1-*2-fed third-instar larvae were reduced by 87.3% and 91.6% respectively, the transcript levels in ds*NAT1-*1- and ds*NAT1-*2-fed fourth-instar larvae were decreased by 88.8% and 77.4% respectively, compared with those in PBS- and ds*egfp*-fed third- and fourth-instar larvae (Figs 4A and 5A).

The consumed foliage areas per day were measured 3 days after the bioassays, the larvae having been fed on PBS-, ds*egfp*-, ds*NAT1-*1-, ds*NAT1-*2-dipped leaves consumed similar amounts of food (Figs 4B, 5B).

The contents of the nineteen proteinogenic amino acids in the bodies and feces of the *LdNAT1* RNAi larvae were measured (Table 2, Tables S2, S3). The contents of cysteine, histidine, isoleucine, leucine, methionine, phenylalanine and serine in the bodies of the *LdNAT1* RNAi hypomorphs were significantly lower than those in the bodies of PBS- and ds*egfp*-fed larvae, whereas the contents of these amino acids in the feces of the *LdNAT1* RNAi hypomorphs were significantly higher (Table 2, Tables S2, S3). These data indicate that knockdown of *LdNAT1* reduces the absorption of the seven amino acids by the larval gut.

### Knockdown of LdNAT1 retards larval growth and impairs pupation

When the larvae were weighed after 3, 4, 5 and 6 days of the initiation of the bioassays, the fresh weights were significantly reduced in the *LdNAT1* RNAi larvae (Figs 4C and 5C).

Exposure to ds*NAT*-immersed foliage significantly delayed larval developing stage. The mean periods of those on water-, ds*egfp-,* ds*NAT1-*1-, ds*NAT1-*2-dipped leaves were 14.0, 13.8, 16.0 and 16.3 days in the larvae that had been treated at the second instar stage, and were 12.0, 12.1, 15.0 and 15.0 days in the larvae having been treated at the third instar stage. In addition, the average periods of 2^nd^, 3^rd^ and 4^th^ instars, and prepupae were further measured. Exposure to ds*NAT1-*1-, ds*NAT1-*2-dipped foliage significantly delayed the developing periods of the fourth-instar and prepupae (Table 3).

Moreover, the pupation rates were significantly decreased in the *LdNAT1* RNAi larvae, compared with those in the PBS- and ds*egfp*-fed larvae (Figs 4D and 5D).

### Knockdown of LdNAT1 disrupts insulin/TOR, JH and 20E signaling pathways

**Figure 6:**
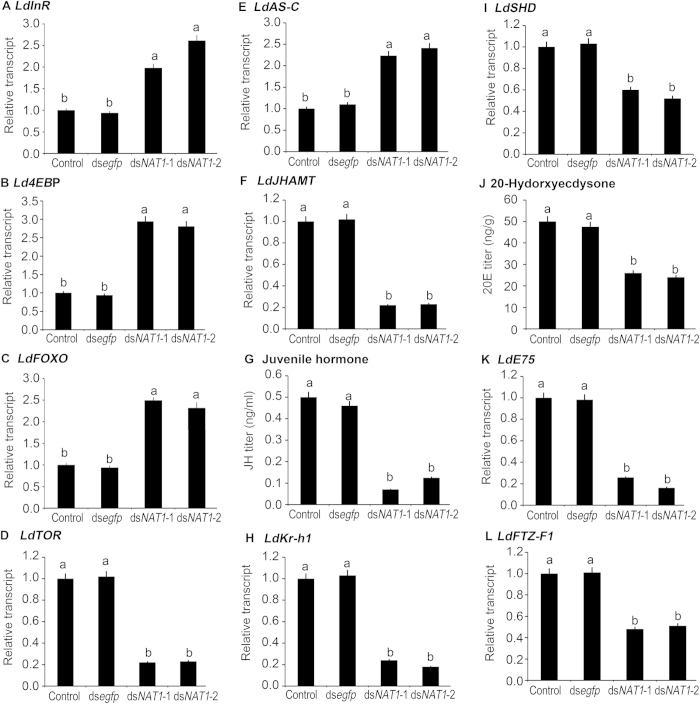
Effects of dietary ingestion of ds*NAT1* (ds*NAT1*-1, ds*NAT1*-2) by the *L. decemlineata* third-instar larvae on insulin/TOR (the left column, A–D), juvenile hormone (the middle column, E–H) and 20-hydroxyecdysone (the right column, I–L) signaling pathways. Relative transcripts are 2^−ΔΔCt^ values (±SE), normalized to the geometrical mean of housekeeping gene expression. Juvenile hormone and 20-hydroxyecdysone titers are tested by a liquid chromatography tandem mass spectrometry system and a liquid chromatography tandem mass spectrometry-mass spectrometry system. Different letters indicate significant difference at P value < 0.05.

InR, 4EBP, FOXO and TOR are core components in insulin/TOR signaling pathway^38^. We sequenced cDNAs encoding InR, 4EBP, FOXO and TOR from *L. decemlineata*. After the initiation of the bioassay for 3 days, the expression levels of the four genes in the fourth-instar alive larvae were tested. *LdInR, LdFOXO* and *Ld4EBP* mRNA levels in ds*NAT1*-1- and ds*NAT1*-2-fed larvae were significantly upregulated. In contrast, *LdTOR* mRNA level was significantly downregulated in the *LdNAT1* RNAi larvae (Fig. 6A–D).

In the *LdNAT1* RNAi hypomorphs, *LdAS-C* expression levels were significantly increased (Fig. 6E), whereas *LdJHAMT* mRNA levels were dramatically reduced (Fig. 6F) and JH titers were significantly decreased (Fig. 6G). As a result, *LdKr-h1* transcript levels were diminished (Fig. 6H).

Finally, ingestion of ds*NAT1*-1 and ds*NAT1*-2 by the third-instar larvae significantly reduced *LdSHD* expression levels (Fig. 6I), decreased 20E titers (Fig. 6J), and diminished the transcript levels of *LdE75* and *LdFTZ-F1-1* (Fig. 6K,L).

## Discussion

In this survey, a putative *LdNAT1* were cloned from *L. decemlineata. Ld*NAT1 had a high amino acid similarity to homologs from other insect species. The phylogenetic result indicated that *Ld*NAT1 was distantly related to other insect NAT1-like proteins. Moreover, *Ld*NAT1 was predicted to have 12 transmembrane domains, which was in agreement with the general structure of transporters in the SLC6 family^11^. It appears that *Ld*NAT1 may be among insect NAT-SLC6 members, and may play a principal role in active absorption and distribution of essential amino acids in *L. decemlineata*.

### Tissue expression of LdNAT1

In insects, amino acids taken up from midgut need to traverse at least three membranes to reach their intracellular site of use: (1) uptake into an epithelial gut cell, (2) basolateral efflux from the gut cell into the hemolymph, and (3) uptake into somatic cell. In general, SLC6 transporters potentially mediate the uptake steps (1) and (3), whereas other SLC members are involved in the efflux step (2)^1^,^39^.

In this survey, tissue expression profiles revealed that *LdNAT1* mRNA levels were high or moderate in the larval gut, and lower in other surveyed tissues. Similarly, *NAT* genes in mammalians, other insect species and nematodes are highly expressed in the apical membranes of the alimentary canal, as well as in other organs with elevated requirements for essential amino acids^1^,^11^,^12^,^13^,^37^,^39^. For example, *MsKAAT1* in *M. sexta*^7^, *AeAAT1*^11^ and *AeNAT5*^14^ in *A. aegypti*, *AgNAT6*^37^ and *AgNAT8*^12^ in *A. gambiae* were highly expressed in the larval guts. Moreover, insect *NAT*s showed unique expression patterns in neurons of the central ganglia and sensory system, which suggest their role as substrate providers for the synthesis of monoamine neurotransmitters^12^,^37^.

Therefore, the tissue expression patterns of *LdNAT1* are compatible with the common idea that NATs function in: (1) active epithelial uptake of amino acids from the lumen of the gut to support systemic amino acid requirements; (2) active uptake of amino acids into specific cells to support their specialized metabolism or growth; and (3) control of the extracellular concentration of neurotransmitter amino acids in the context of synaptic transmission^39^.

### JH triggers whereas 20E represses the expression of LdNAT1

Essential amino acids are absorbed by midgut in insects^11^,^12^,^13^. In *L. decemlineata* midgut, several cysteine proteases such as intestain A through E have been reported to digest foliage protein^40^,^41^. Vacuolar H^+^-ATPases energize Na^+^ and/or K^+^/H^+^ antiport^42^ to actively transport Na^+^/K^+^ from the hemolymph into the midgut lumen. Na^+^/K^+^ ions are expected to subsequently trigger Na^+^/K^+^-dependent NATs to absorb amino acids in *L. decemlineata*, similar to the NAT-SLC6 members reported in other insect species^43^.

In some insect species, genes encoding proteases, ATPase subunits and some NAT members are regulated by JH/20E. In *Spodoptera litura* larvae, for example, a putative serine protease gene *Slctlp2* was induced by JH III but not 20E^44^. In *Helicoverpa armigera*, the expression of a trypsin-like serine protease gene *HaTLP* was upregulated by a JH analog methoprene and downregulated by 20E *in vivo*^45^. In *A. aegypti*, the transcription of a serine-type protease gene *JHA15* is activated by JH in the newly emerged female adults^46^. Moreover, the expression of V-ATPase subunit genes were repressed by 20E in *M. sexta*^47^. Furthermore, two SLC7 genes, *JhI-21* and *minidiscs* (*mnd*), were JH-inducible in *D. melanogaster*^48^. However, whether *NAT-SLC6* genes in insects respond to JH/20E remains unproven.

In several insect species, JH peaks and troughs are observed at each ecdysis. In a termite *Cryptotermes secundus*, for example, within each instar the JH titer rose shortly before or right after the molt, and then dropped sharply^49^. Similar phenomenon was observed in *L. decemlineata*^27^ and *M. sexta*^47^. In this study, temporal expression patterns indicate that *LdNAT1* transcript level appears to be positively correlated with the titer of JH. Thus, we determined whether the correlation had any biological significance. As expected, we discovered that either JH or Pyr induced the expression of *LdNAT1* in an *in vivo* bioassay. Moreover, we found that knockdown of *LdAS-C* to increase JH titer^27^ activated *LdNAT1* expression. Conversely, silencing *LdJHAMT* to inhibit JH biosynthesis reduced *LdNAT1* transcript in *L. decemlineata* final instar larvae.

Temporal expression patterns also imply that 20E inhibits *LdNAT1* transcription. As expected, we found that *LdNAT1* expression was dramatically decreased in *L. decemlineata* specimens having ingested 20E or an ecdysteroid agonist Hal. Conversely, a decrease in 20E in the *LdSHD* RNAi hypomorphs activated the expression of *LdNAT1*. Thus, 20E represses the transcription of *LdNAT1* in *L. decemlineata.* In holometabolous insects such as *D. melanogaster*, 20E signal directly induces transcription of early 20E-response genes such as *DmBR-C*, *DmE74A* and *DmE75A*, and upregulates an early-late gene *DmHR3* during larval-pupal transition^50^. *Dm*HR3 then induces *DmβFTZ-F1* expression in mid-prepupae^51^. In this survey, knockdown of either *LdE75* or *LdFTZ-F1* reduced 20E titer, as our previously reported results^29^, the expression levels of *LdNAT1* in both *LdE75* and *LdFTZ-F1* RNAi larvae were significantly increased. Therefore, our results suggested that *LdNAT1* repression required complete 20E signaling pathway.

In response to JH and 20E, *LdNAT1* transcription is activated right after the molt, and is repressed just before the molt. Thus, its protein may function in absorption of amino acids at the mid instar stage when *L. decemlineata* larvae are actively feeding.

### Involvement of LdNAT1 in uptake of neutral amino acids

*Xenopus* oocyte-expressed insect transporters, such as *M. sexta Ms*KAAT1^5^ and *Ms*CATCH1^10^, *A. aegypti Aa*NAT1^11^ and *D. melanogaster Dm*NAT1^13^, shared relatively broad substrate spectra. *Ms*KAAT1 absorbed phenylalanine, tryptophan, isoleucine, leucine, valine, methionine and alanine^5^,^7^,^8^,^9^, whereas *Ms*CAATCH1 preferred threonine in the presence of K^+^ but preferred proline in the presence of Na^+^ 10. *Ae*AAT1 from *A. aegypti* had notable apparent affinities and transport velocities for phenylalanine, cysteine, histidine, alanine, serine and methionine^11^. *Dm*NAT1 in *D. melanogaster* transported threonine, isoleucine, leucine, valine, histidine, phenylalanine, tyrosine, tryptophan, methionine, cysteine, alanine, proline, serine, asparagine and glycine, with virtually equal apparent affinities and transport velocities^13^. In contrast, several Dipteran NAT-SLC6 members showed narrow specialization for absorption of essential amino acids^12^,^14^,^37^. However, their orphan orthologs are absent in *D. melanogaster* or outside Diptera species^14^.

In this survey, by comparing the compositions of nineteen proteinogenic amino acids (tryptophan is undetectable) in potato foliage, and in bodies and feces of the day 1 third- and fourth-instar larvae, we found that a total of 11 amino acids (cysteine, histidine, lysine, methionine, serine, tyrosine, valine, glutamate, isoleucine, leucine, phenylalanine) may be actively absorbed by *L. decemlineata* larval gut. In the *LdNAT1* RNAi larvae, the contents of cysteine, histidine, isoleucine, leucine, methionine, phenylalanine and serine in the bodies were significantly lower, whereas the contents of these amino acids in the feces were significantly higher, compared with those in the bodies and feces of PBS- and ds*egfp*-fed larvae. Therefore, *Ld*NAT1 in *L. decemlineata* appears an SLC6-NAT transporter belonging to the B(0) system, like most of its homologs in Dipteran and Lepidopteran insect species^5^,^10^,^11^,^13^.

However, among 11 amino acids being suggested active absorption by the larval gut in *L. decemlineata* in this survey, lysine, tyrosine, valine and glutamate can not be absorbed by *Ld*NAT1. This indicates that there are other functional NATs in *L. decemlineata*. Consistent with our results, a total of 9 SLC families are present in insects out of 10 SLC families participating in mammalian amino acid transport 1. Work is in progress to identify these transporters.

### Knockdown of LdNAT1 impaired larval development

Silencing *LdNAT1* caused obvious negative effects: larval growth was retarded; development period was lengthened, and pupation was impaired. Similarly, RNAi of *Ae*NAT5 increased larval mortality during ecdysis and dramatically suppressed adult emergence in *A. aegypti*^14^. Moreover, SLC6 was prominent in bacteria and archaea, and often served in environmental absorption of tryptophan, phenylalanine, tyrosine and methionine. Depriving the organism of such amino acids, or knocking out bacterial SLC6 transporters, limited the exponential growth of bacterial populations^52^,^53^,^54^,^55^,^56^. In *Caenorhabditis elegans*, *snf-5* encodes a SLC6 family NAT. A loss-of-function mutation in *snf-5* increased dauer formation and reduced dauer maintenance upon starvation^39^.

In insect, nutritional deprivation inhibited insulin/TOR pathway^38^ to modulate insect growth and metamorphosis^22^,^23^,^24^. Thus, we measured the expression levels of *LdInR*, *Ld4EBP*, *LdFOXO* and *LdTOR*. As expected, the transcription of *LdInR*, *Ld4EBP* and *LdFOXO* was upregulated in *L. decemlineata*. In contrast, the mRNA level of *LdTOR* was reduced.

Our results further revealed that exposure to ds*NAT1-*1-, ds*NAT1-*2-dipped foliage significantly delayed the developing periods of the fourth-instar and prepupae, whenever we treated the larvae at the second-instar or at the third-instar stages. Thus, NAT is required for dietary intake of amino acids supporting growth and development to critical weight at the fourth (final) instar stage. Moreover, we further discovered that knockdown of *LdNAT1* reduced the mRNA level of an ecdysteroidogenesis gene *LdSHD*, decreased 20E titer, and lowered the expression of two 20E-response gene *LdE75* and *LdFTZ-F1*^29^. Furthermore, silencing *LdNAT1* increased the mRNA level of *LdAS-C*, reduced the expression of a JH biosynthesis gene *LdJHAMT*, decreased JH titer, and diminished the transcription of a JH early-inducible gene *LdKr-h1* in *L. decemlineata* larvae.

**Figure 7:**
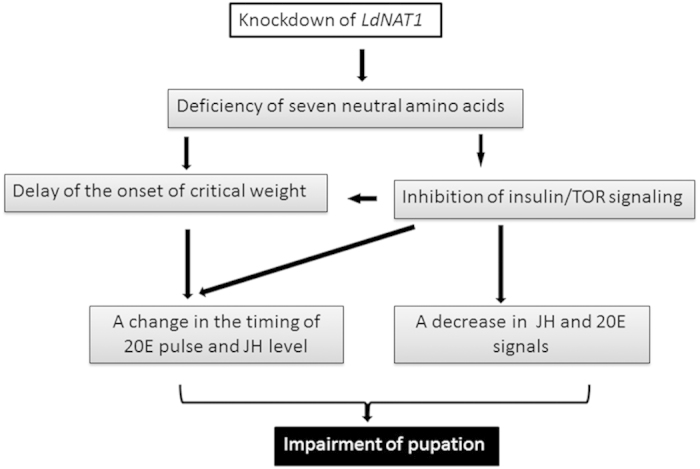
A model for *LdNAT1* knockdown on *Leptinotarsa* larval-pupal metamorphosis. Knockdown of *LdNAT1* results in the deficiency of seven neutral amino acids, i.e., cysteine, histidine, isoleucine, leucine, methionine, phenylalanine and serine. This delays the onset of critical weight and inhibits insulin/TOR signaling. The onset of critical weight and the inhibited insulin/TOR signaling may change the timing of the 20E pulse and JH level in the final instar larvae. Moreover, the reduced insulin/TOR signaling may also decrease 20E and JH signals. Thus, the growth is retarded and the pupation is impaired in the *LdNAT1* RNAi hypomorphs.

There are at least two alternative, but not mutually exclusive, explanations for the results. Firstly, delay of the onset of critical weight and reduced insulin/TOR signaling change the timing of 20E pulse and the level of circulating JH in the *LdNAT1* RNAi hypomorphs. Alternatively, the reduced insulin/TOR signaling decreases circulating 20E and JH^22^,^23^,^24^. Disturbed 20E and JH signals subsequently delay larval development and impaired pupation. Accordingly, a model for the influence of knockdown of *LdNAT1* on the larval-pupal metamorphosis is proposed in *L. decemlineata* (Fig. 7).

## Additional Information

**How to cite this article**: Fu, K.-Y. *et al.* Knockdown of a *nutrient amino acid transporter* gene *LdNAT1* reduces free neutral amino acid contents and impairs *Leptinotarsa decemlineata* pupation. *Sci. Rep.* **5**, 18124; doi: 10.1038/srep18124 (2015).

## Supplementary Material

Supplementary Information

## Figures and Tables

**Table 1:** The compositions of amino acid (%) in foliage, the bodies and feces of the day 1 third- and fourth-instar larvae.

		The third-instar larvae		The fourth-instar larvae	
Foliage		Bodies	Feces	Bodies	Feces
A	8.70 ± 0.41 a	7.75 ± 0.43 a	8.58 ± 0.52 a	7.60 ± 0.37 ab	6.45 ± 0.54 b
R	4.89 ± 0.27 a	4.42 ± 0.33 a	4.44 ± 0.35 a	4.50 ± 0.29 a	3.34 ± 0.26 b
D	1.23 ± 0.11 a	0.91 ± 0.05 a	0.95 ± 0.07 a	0.92 ± 0.08 a	0.71 ± 0.06 a
N	9.79 ± 0.81 a	7.47 ± 0.54 b	8.58 ± 0.43 a	8.25 ± 0.74 ab	10.81 ± 1.12 a
C	0.25 ± 0.02 b	1.36 ± 0.12 a	0.08 ± 0.00 c	0.88 ± 0.07 ab	0.06 ± 0.00 c
E	0.96±0.10 ab	1.38 ± 0.11 a	1.06 ± 0.13 a	1.34 ± 0.12 a	0.80 ± 0.09 b
Q	9.73 ± 0.85 a	12.33 ± 1.02 a	9.72 ± 0.81 a	11.65 ± 1.12 a	12.79 ± 1.23 a
G	9.62 ± 0.81 c	9.04 ± 0.75 c	18.07 ± 1.52 b	9.16 ± 1.04 c	24.60 ± 2.33 a
H	1.92 ± 0.15 b	2.56 ± 0.14 a	1.58 ± 0.12 b	2.74 ± 0.16 a	1.19 ± 0.09 c
I	4.71 ± 0.22 a	5.09 ± 0.23 a	5.22 ± 0.36 a	5.04 ± 0.42 a	3.92 ± 0.28 b
L	8.87 ± 0.52 a	7.17 ± 0.48 a	6.88 ± 0.54 ab	8.35 ± 0.57 a	5.94 ± 0.34 b
K	6.49 ± 0.33 a	7.62 ± 0.35 a	2.81 ± 0.23 b	7.52 ± 0.48 a	2.93 ± 0.23 b
M	1.35 ± 0.12 a	0.21 ± 0.01 b	0.01±0.00 c	0.20 ± 0.03 b	0.03 ± 0.00 c
F	4.22 ± 0.34 a	3.38 ± 0.30 ab	3.91 ± 0.20 a	3.37 ± 0.18 ab	2.94 ± 0.18 b
P	6.42 ± 0.53 a	6.26 ± 0.50 a	7.39 ± 0.64 a	5.63 ± 0.59 a	5.56 ± 0.53 a
S	5.59 ± 0.34 a	6.81 ± 0.42 a	4.49 ± 0.35 b	6.79 ± 0.32 a	4.15 ± 0.27 b
T	5.32 ± 0.34 bc	4.65 ± 0.42 c	8.31 ± 0.72 a	4.62 ± 0.38 c	6.26 ± 0.46 b
Y	3.07 ± 0.22 a	3.19 ± 0.17 a	2.61 ± 0.14 b	3.23 ± 0.21 a	1.96 ± 0.15 c
V	6.79 ± 0.37 b	8.31 ± 0.42 a	5.22 ± 0.25 c	8.12 ± 0.45 a	5.45 ± 0.30 c

Note: A, alanine; R, arginine; D, aspartic acid; N, asparagine; C, cysteine; E, glutamate; Q, glutamine; G, glycine; H, histidine; I, isoleucine; L, leucine; K, lysine; M, methionine; F, phenylalanine; P, proline; S, serine; T, threonine; Y, tyrosine; V, valine. Data within each line are subjected to arc-sine transformation and analyzed by ANOVAs followed by the Tukey-Kramer test. Different letters indicate significant difference at P value < 0.05.

**Table 2:** The contents of seven amino acids in the bodies and feces of the *Leptinotarsa decemlineata* larvae fed on PBS, ds*egfp*, ds*NAT1-*1 and ds*NAT1-*2-immersed foliage for 3 days.

Amino acid	Origin	Concentration (μmol/g)			
		PBS	ds*egfp*	ds*NAT1-*1	ds*NAT1-*2
The third-instar larvae					
C	Bodies	11.12 ± 0.51 a	13.12 ± 0.84 a	3.21 ± 0.21 b	2.67 ± 0.14 b
	Feces	0.25 ± 0.02 b	0.33 ± 0.03 b	2.34 ± 0.14 a	3.15 ± 0.13 a
H	Bodies	20.88 ± 1.24 a	20.02 ± 1.11 a	10.70 ± 0.52 b	11.17 ± 0.47 b
	Feces	4.69 ± 0.20 b	4.06 ± 0.28 b	8.84 ± 0.41 a	7.87 ± 0.52 a
I	Bodies	41.49 ± 2.22 a	42.16 ± 3.04 a	26.77 ± 1.29 b	28.13 ± 1.38 b
	Feces	15.41 ± 0.84 b	12.68 ± 0.61 b	27.22 ± 1.15 a	29.39 ± 1.74 a
L	Bodies	58.48 ± 2.41 a	58.76 ± 1.98 a	32.45 ± 2.07 b	34.68 ± 2.15 b
	Feces	20.31 ± 1.06 b	19.75 ± 1.19 b	37.55 ± 1.24 a	33.17 ± 2.03 a
M	Bodies	1.74 ± 0.38 a	4.26 ± 0.25 a	0.64 ± 0.04 b	0.13 ± 0.01 b
	Feces	0.02 ± 0.00 b	0.03 ± 0.00 b	0.31 ± 0.02 a	0.25 ± 0.02 a
F	Bodies	27.55 ± 1.07 a	28.64 ± 1.13 a	19.20 ± 1.23 b	15.73 ± 1.19 b
	Feces	11.56 ± 0.81 b	9.62 ± 0.57 b	23.69 ± 1.04 a	18.96 ± 1.07 a
S	Bodies	55.50 ± 2.11 a	55.86 ± 4.08 a	41.88 ± 2.58 b	34.26 ± 2.14 b
	Feces	13.27 ± 1.01 b	11.67 ± 0.72 b	31.84 ± 1.22 a	29.62 ± 1.57 a
The fourth-instar larvae					
C	Bodies	6.89 ± 0.51 a	10.50 ± 0.67 a	2.10 ± 0.16 b	0.25 ± 0.02 c
	Feces	0.25 ± 0.02 b	0.25 ± 0.02 b	4.58 ± 0.37 a	7.70 ± 0.51 a
H	Bodies	21.32 ± 0.71 a	19.86 ± 0.54 a	9.44 ± 0.35 b	10.50 ± 0.49 b
	Feces	4.69 ± 0.57 b	4.66 ± 0.41 b	8.64 ± 0.37 a	12.06 ± 0.61 a
I	Bodies	39.21 ± 1.13 a	45.54 ± 2.02 a	21.79 ± 1.51 b	20.38 ± 1.44 b
	Feces	15.40 ± 0.87 b	13.72 ± 0.74 b	30.73 ± 1.57 a	27.28 ± 1.63 a
L	Bodies	65.00 ± 3.54 a	63.12 ± 4.13 a	38.40 ± 2.33 b	37.77 ± 1.79 b
	Feces	23.30 ± 1.47 b	26.91 ± 1.56 b	46.79 ± 3.64 a	48.52 ± 4.11 a
M	Bodies	1.60 ± 0.11 a	1.54 ± 0.08 a	0.65 ± 0.02 b	0.41 ± 0.01 b
	Feces	0.15 ± 0.01 b	0.40 ± 0.02 b	2.64 ± 0.15 a	1.51 ± 0.11 a
F	Bodies	26.25 ± 1.47 a	29.72 ± 1.55 a	11.51 ± 0.71 b	14.34 ± 0.57 b
	Feces	11.55 ± 0.57 b	12.97 ± 0.69 b	22.34 ± 1.21 a	24.58 ± 1.54 a
S	Bodies	52.88 ± 3.74 a	59.52 ± 3.69 a	26.64 ± 1.27 b	28.63 ± 1.34 b
	Feces	16.27 ± 0.79 b	15.34 ± 0.85 b	31.11 ± 1.45 a	42.19 ± 1.85 a

Free amino acid contents in the bodies and feces are analyzed with a Beckman 6300 Amino Acid Analyzer. Difference of amino acid content within each line is analyzed by ANOVA followed by the Tukey-Kramer test. Data that do not share the same letters are significantly different at P values of 0.05.

**Table 3:** The developing stage of *L. decemlineata* surviving larvae subjected to dietary dsRNA introduction.

Larval instar	2^nd^	3^rd^	4^th^	Prepupae	
Initiation of the bioassay at the early second instar stage					
CK	2.0 ± 0.1 a	2.1 ± 0.2 a	4.1 ± 0.2 a	5.8 ± 0.3 a	14.0 ± 0.4 a
ds*egfp*	2.2 ± 0.2 a	2.0 ± 0.1 a	4.0 ± 0.2 a	5.6 ± 0.3 a	13.8 ± 0.5 a
ds*NAT1*-1	2.1 ± 0.1 a	2.1 ± 0.2 a	5.1 ± 0.2 b	6.7 ± 0.4 b	16.0 ± 0.6 b
ds*NAT1*-2	2.1 ± 0.1 a	2.2 ± 0.2 a	5.2 ± 0.3 b	6.8 ± 0.4 b	16.3 ± 0.5 b
Initiation of the bioassay at the early third instar stage					
CK		2.0 ± 0.1 a	4.0 ± 0.2 a	6.0 ± 0.2 a	12.0 ± 0.3 a
ds*egfp*		2.1 ± 0.2 a	4.2 ± 0.1 a	5.8 ± 0.4 a	12.1 ± 0.4 a
ds*NAT1*-1		2.0 ± 0.1 a	5.4 ± 0.2 b	7.6 ± 0.3 b	15.0 ± 0.5 b
ds*NAT1*-2		2.2 ± 0.1 a	5.3 ± 0.2 b	7.5 ± 0.3 b	15.0 ± 0.4 b

The larval growth is checked at 4-hr intervals. See text for detail explanation for the identification of instars and prepupae. The data are given as means ± SE, and are subjected one-way ANOVA and followed by the Tukey–Kramer test. Means on the same column followed by the same letters are not significantly different at P < 0.05.
