# Supplementary material for: Knockdown of a nutrient amino acid transporter gene LdNAT1 reduces free neutral amino acid contents and impairs Leptinotarsa decemlineata pupation
Source: Sci Rep. 2015 Dec 11;5:18124. doi: 10.1038/srep18124 (PMC4676043; doi:10.1038/srep18124)
Supplement: Supplementary Information [file srep18124-s1.pdf]

Knockdown of *a nutrient amino acid transporter* gene *LdNAT1* reduces free neutral amino acid contents and impairs *Leptinotarsa decemlineata* pupation

Kai-Yun Fu[1], Wen-Chao Guo[2], Tursun Ahmat[2], Guo-Qing Li [1] *

1. Education Ministry Key Laboratory of Integrated Management of Crop Diseases and Pests, College of Plant Protection, Nanjing Agricultural University, Nanjing 210095, China
2. Department of Plant Protection, Xinjiang Academy of Agricultural Sciences; Urumqi 830091, China

Table S1. Primers used for RT-PCR, 5' or 3'RACE, dsRNA synthesis and qRT-PCR

Fragment name	Forward primer(5'-3')	Reverse primer(5'-3')
**RT-PCR**		
LdNAT1	CTTCCTTGGACCGAGTGT	AATGTGATCCGCATACCT
Ld4EBP	GTAAAGGGTTACAGTGTCAAG	AAACGGCAGTTAGAAGTAGA
LdINR	CGTTTTCAATACCAACTTCAGC	CGACTCCTGCGGGACTATG
LdTOR	AACAATGTTACGAGGAGTCTGG	TCGTGAGTTGTATTTGCCTGT
LdFOXO	AACAATGTTACGAGGAGTCTGG	TCGTGAGTTGTATTTGCCTGT
LdE75A	ATAAAGTTGAGCCGATGTTG	ATCTGGAGTTGGGATTGC
LdE75B	TACCCCTATGGAGGAAGAG	GTGATACAAACAGGCGAGA
LdE75C	AAAACTGAGCCCAACGAG	CAACGGCCTTATCCAAAG
**RACE**		
LdNAT1 5'-G		CCTTTCACGGCATTGTCATTCTCA
Ld4EBP 5'-G		TGGCAAGATGCTCAGGGATGTG
LdINR 5'-G		CAATCTGCTGAGCCTTGTTGTAGTGG
LdNAT1 3'-G	TGGAGAATGTCGTGGGGACTCAT	
Ld4EBP 3'-G	CACATCCCTGAGCATCTTGCCATT	
LdINR 3'-G	AGAGCATCACGATCCACCTCTTCAG	
LdE75A 5'-G		CGGTATTTCTGAGGCCGGGACGAT
LdE75A 5'-N		TCGACTACACCACGTATAGCTGGGGAAA
**ORF verification**		
LdNAT1	GGAAAATCAGGCGTAAAA	AGTAATTGAAAACGACGC
Ld4EBP	CATGCCTGAATCTCCTAA	AACAGATCAAGTCATCCC

LdINR	TTGCCTGGCTTATTTACC	TTATCGAGCTGTTTGCTT
LdE75A	GCAGTTTGGTCGTGATAC	AGAGTGCTAGGGATTGGT
LdE75B	TTGTAGATAGGTTGAGGGAG	AGAGTGCTAGGGATTGGT
LdE75C	GTGCGTGATTGGCCTAGG	TTCTGGTCATGGCGGAAG
**dsRNA synthesis**		
dsLdNAT1-1	TTTCATACCCAGAAGCTA	CCATGCTGTGTTTTCTGTCA
dsLdNAT1-2	GGACTCGGAATACCAGAT	GGAAACTATTATCGCATCA
dsSHD	TTGGTGTTCCTCCTCTACCTTG	TGGCCTTGTAACTTCTACGTCTAT
dsE75	TCCCAAGAGAGAAAAAGCG	GGCAAGCAAGGAGAATCC
dsFTZ-F1	TCTGCTGAGTTTGGGGTT	GTGCTTGTGGTAGAGGTGTT
dsAS-C	GGCATCTGGGAGAAAATAG	GAAGTAGCAGGCTCTGAATCT
dsJHAMT	GGACAAGCCCGACTTATACTC	GCGAACTCCACCTCATCAA
dsegfp	AAGTTCAGCGTGTCCG	CTTGCCGTAGTTCCAC
**qRT-PCR**		
qLdNAT1	CCGGCACAGGCTTAGCATTCATTT	AGAGCTATCAGGCTTCCAACTCCT
qLdInR	TCTCAAGCGAATCAGAGGTG	GTTACGCCAATCCCAAAGTT
qLd4EBP	TGCAGGGAGATCTCAACAAA	CAGAGAAGATCAACAAGCAGGA
qLdTOR	GGCTGAAATATGCTGCGTTA	AGGAGAAACCATTGGCAAAC
qLdFOXO	CGCGTAAACTACTGCGTCAT	GCACTGAACCACAGATCCAC
qLdAS-C	ATGCAAGGCTTTCTCCATCT	TTGATTGTTGTCCGGGTCTA
qLdJHAMT	GGAAGTGGAGATGGCAAGTT	CTACCAACGAGTTTCCCGAT
qLdKr-h1	ATCAAGTGCCATTCCGAAAGCAA	ATGTCGGAATCATAGCTGACGGGT
qLdSHD	GGCCTGAACTGGTTTGCAT	GGCAAGAATAACCGAGGAAGAG
qLdE75	CAAGACCAGTGGGAAATCAG	ATTGTCGCCATCCTGAGATT
qLdFTZ-f1	CAAATGAAGCTTCTCCAGCA	TATGAAGGGTGGTTTCGTCA
qLdRP4	AAAGAAACGAGCATTGCCCTTCCG	TTGTCGCTGACACTGTAGGGTTGA
qLdRP18	TAGAATCCTCAAAGCAGGTGGCGA	AGCTGGACCAAAGTGTTTCACTGC
qLdARF1	CGGTGCTGGTAAAACGACAA	TGACCTCCCAAATCCCAAAC
qLdARF4	GTGCTCGTGAACCATGTGAA	AACCTCCAATCCCTCGTGAA

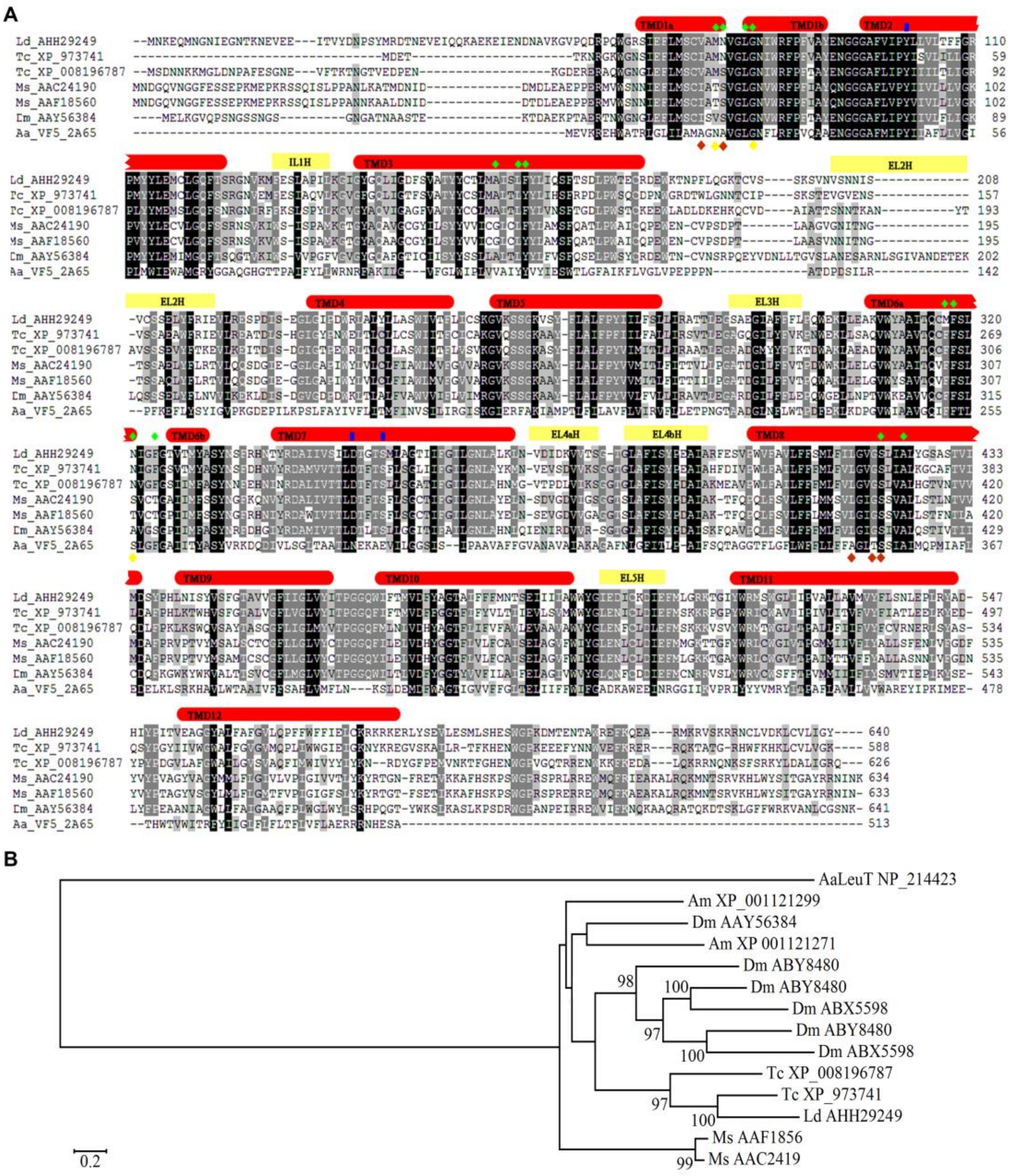

Figure S1. Multiple alignment (A) and phylogenetic analysis (B) of a putative NAT1 from *Leptinotarsa decemlineata*. The amino acid sequence of *Ld*NAT1 (Ld_AHH29249) is aligned with those from *Tribolium castaneum* (Tc, XP_973741; XP_008196787), *Manduca sexta* (Ms, AAC24190; AAF18560), *Drosophila melanogaster* (Dm, AAY56384), and *Aedes aegypti* (Aa, VE5_2A65). Increasing background intensity indicates an increase in sequence similarity. Red color bars above the sequences are transmembrane domains (TMDs). Green diamonds indicate substrate-binding sites, red and brown rhombuses are the first and second sodium ion-binding sites, respectively, in other SLC6 members. For phylogenetic analysis, NAT-like representatives originate from 6 insect species. Bootstrap values (1000 replicates) are displayed by the nodes. The genetic distance is drawn to scale.

Table S2. Content of hydrolytic amino acids in the potato foliage (μmol/g)

Amino acid	Content
Alanine (Ala)	45.57±4.16
Arginine (Arg)	25.63±2.05
Aspartic acid (Asp)	6.44±3.86
Asparagine (Asn)	51.34±4.54
Cysteine (Cys)	1.31±0.08
Glutamate (Glu)	5.03±0.31
Glutamine (Gln)	50.97±3.98
Glycine (Gly)	50.39±4.25
Histidine (His)	10.08±0.54
Isoleucine (Ile)	24.69±1.31
Leucine (Leu)	46.45±3.84
Lysine (Lys)	34.01±2.57
Methionine (Met)	7.07±0.71
Phenylalanine (Phe)	22.11±1.74
Proline (Pro)	33.64±2.41
Serine (Ser)	29.29±2.17
Threonine (Thr)	27.86±1.34
Tyrosine (Tyr)	16.11±1.02
Valine (Val)	35.59±2.51
Total	523.58±45.33

Hydrolytic amino acids in the foliage were extracted by acid hydrolysis method, and analyzed with a Beckman 6300 Amino Acid Analyzer.

Table S3. Contents of free amino acids in the body and feces of *Leptinotarsa decemlineata* third-instar larvae fed on PBS, ds*egfp*, ds*NAT1*-1 and ds*NAT1*-2-immersed foliage for 3 days

Amino acid	Origin	Concentration (μmol/g)			
		PBS	ds*egfp*	ds*NAT1*-1	ds*NAT1*-2
Alanine (Ala)	Body	59.19±4.16 a	73.79±5.69 a	63.73±4.32 a	65.08±3.83 a
	Feces	25.32±2.71 a	41.18±3.44 a	40.91±3.58 a	61.17±6.79 a
Arginine (Arg)	Body	35.07±1.69 a	35.09±3.45 a	32.89±3.07 a	35.79±2.67 a
	Feces	13.11±0.81 a	17.32±1.13 a	24.95±1.27 a	23.35±1.06 a
Aspartic acid (Asp)	Body	7.18±0.49 a	8.73±0.54 a	7.75±0.43 a	7.93±0.50 a
	Feces	2.81±0.18 a	4.74±0.35 a	3.97±0.36 a	4.56±0.29 a
Asparagine (Asn)	Body	64.23±4.67 a	80.11±5.81 a	70.24±4.48 a	72.81±5.07 a
	Feces	42.38±2.25 a	25.47±2.04 b	36.49±3.16 ab	48.37±3.57 a
Cysteine (Cys)	Body	6.89±0.51 aA	10.50±0.67 aA	2.10±0.16 bB	0.25±0.02 cB
	Feces	0.25±0.02 B	0.25±0.02 B	4.58±0.37 A	7.70±0.51 A
Glutamate (Glu)	Body	10.43±0.81 a	11.95±1.03 a	11.74±0.71 a	11.79±0.54 a
	Feces	3.15±0.34 a	5.98±0.41 a	3.77±0.24 a	5.41±0.40 a
Glutamine (Gln)	Body	90.67±5.09 a	108.64±8.34 a	100.24±7.67 a	102.47±8.34 a
	Feces	50.17±3.77 a	30.47±2.23 a	50.19±4.12 a	34.62±1.38 a
Glycine (Gly)	Body	71.31±6.29 a	86.59±4.98 a	80.10±5.08 a	82.39±4.96 a
	Feces	96.44±8.11 a	109.74±5.08 a	110.32±9.16 a	90.72±5.13 a
Histidine (His)	Body	21.32±0.71 a	19.86±0.54 a	9.44±0.35 b	10.50±0.49 b
	Feces	4.69±0.57 b	4.66±0.41 b	8.64±0.37 a	12.06±0.61 a
Isoleucine (Ile)	Body	39.21±1.13 a	45.54±2.02 a	21.79±1.51 b	20.38±1.44 b
	Feces	15.40±0.87 b	13.72±0.74 b	30.73±1.57 a	27.28±1.63 a
Leucine (Leu)	Body	65.00±3.54 a	63.12±4.13 a	38.40±2.33 b	37.77±1.79 b
	Feces	23.30±1.47 b	26.91±1.56 b	46.79±3.64 a	48.52±4.11 a
Lysine (Lys)	Body	58.53±4.17 a	63.41±7.79 a	53.75±4.81 a	56.47±6.81 a
	Feces	11.51±1.12 a	15.76±1.06 a	15.27±1.14 a	11.12±1.03 a
Methionine (Met)	Body	1.60±0.11 a	1.54±0.08 a	0.65±0.02 b	0.41±0.01 b
	Feces	0.15±0.01 b	0.40±0.02 b	2.64±0.15 a	1.51±0.11 a
Phenylalanine (Phe)	Body	26.25±1.47 a	29.72±1.55 a	11.51±0.71 b	14.34±0.57 b
	Feces	11.55±0.57 b	12.97±0.69 b	22.34±1.21 a	24.58±1.54 a
Proline (Pro)	Body	43.85±4.17 a	59.87±5.44 a	66.94±6.13 a	57.02±5.84 a
	Feces	21.83±2.23 b	31.22±2.47 ab	41.94±3.49 a	44.33±3.12 a
Serine (Ser)	Body	52.88±3.74 a	59.52±3.69 a	26.64±1.27 b	28.63±1.34 b
	Feces	16.27±0.79 b	15.34±0.85 b	31.11±1.45 a	42.19±1.85 a
Threonine (Thr)	Body	35.95±2.57 a	41.81±3.39 a	41.38±4.01 a	43.73±3.85 a
	Feces	24.54±1.84 a	20.42±1.15 a	24.88±1.59 a	23.70±1.82 a
Tyrosine (Tyr)	Body	25.19±1.48 a	25.60±1.81 a	25.93±1.94 a	25.21±1.76 a
	Feces	7.71±0.71 b	10.66±0.83 ab	14.21±0.91 a	19.08±1.23 a
Valine (Val)	Body	63.18±6.02 a	73.97±6.79 a	76.07±7.05 a	66.18±6.67 a
	Feces	21.40±1.84 b	32.70±2.33 ab	30.69±3.14 ab	43.05±4.17 a
Total	Body	777.93±52.58 a	899.36±63.44 a	711.29±56.81 a	719.15±61.53 a
	Feces	391.98±29.48 a	419.91±31.21 a	554.42±27.68 a	593.32±29.24 a

Free amino acid contents are analyzed with a Beckman 6300 Amino Acid Analyzer. Difference of the content of each amino acid among treatments is analyzed by ANOVA followed by the Tukey-Kramer test. Data that do not share the same lowercase or uppercase letter are significantly different at P values of 0.05 or 0.01.

Table S4. Contents of free amino acids in the body and feces of *Leptinotarsa decemlineata* fourth-instar larvae fed on PBS, ds*egfp*, ds*NAT1*-1 and ds*NAT1*-2-immersed foliage for 3 days

Amino acid	Origin	Concentration (μmol/g)			
		PBS	ds*egfp*	ds*NAT1*-1	ds*NAT1*-2
Alanine (Ala)	Body	63.16±4.33 a	64.05±5.14 a	58.01±3.03 a	60.39±3.81 a
	Feces	25.32±1.57 a	23.65±2.51 a	28.09±1.64 a	39.34±2.09 a
Arginine (Arg)	Body	36.06±2.17 a	34.68±2.05 a	30.51±1.83 a	31.43±1.14 a
	Feces	13.11±0.52 a	11.75±1.42 a	14.63±0.97 a	13.39±0.76 a
Aspartic acid (Asp)	Body	7.49±0.31 a	8.14±0.53 a	7.35±0.40 a	7.52±0.57 a
	Feces	2.81±0.20 a	2.61±0.16 a	3.44±0.17 a	4.23±0.32 a
Asparagine (Asn)	Body	60.93±4.12 a	70.83±5.73 a	66.14±3.31 a	68.41±4.15 a
	Feces	25.33±1.10 b	24.52±0.57 b	41.70±2.13 a	38.54±1.98 a
Cysteine (Cys)	Body	11.12±0.51 A	13.12±0.84 A	3.21±0.21 B	2.67±0.14 B
	Feces	0.25±0.02 B	0.33±0.03 B	2.34±0.14 A	3.15±0.13 A
Glutamate (Glu)	Body	11.28±0.88 a	11.98±1.05 a	10.04±0.94 a	10.76±0.67 a
	Feces	3.15±0.24 a	2.96±0.22 a	4.12±0.35 a	4.76±0.26 a
Glutamine (Gln)	Body	100.52±8.36 a	98.41±7.33 a	90.57±8.21 a	97.53±7.66 a
	Feces	28.71±1.24 a	26.44±2.12 a	35.72±3.56 a	40.33±3.12 a
Glycine (Gly)	Body	73.69±6.24 a	76.52±3.32 a	71.10±5.85 a	78.35±6.43 a
	Feces	53.33±3.51 a	83.09±6.57 a	92.55±5.33 a	115.72±8.41 a
Histidine (His)	Body	20.88±1.24 a	20.02±1.11 a	10.70±0.52 b	11.17±0.47 b
	Feces	4.69±0.20 b	4.06±0.28 b	8.84±0.41 a	7.87±0.52 a
Isoleucine (Ile)	Body	41.49±2.22 a	42.16±3.04 a	26.77±1.29 b	28.13±1.38 b
	Feces	15.41±0.84 b	12.68±0.61 b	27.22±1.15 a	29.39±1.74 a
Leucine (Leu)	Body	58.48±2.41 a	58.76±1.98 a	32.45±2.07 b	34.68±2.15 b
	Feces	20.31±1.06 b	19.75±1.19 b	37.55±1.24 a	33.17±2.03 a
Lysine (Lys)	Body	62.09±3.31 a	61.05±3.54 a	57.71±4.41 a	52.33±3.96 a
	Feces	8.30±0.64 a	11.01±0.59 a	13.00±0.81 a	10.11±0.90 a
Methionine (Met)	Body	1.74±0.38 a	4.26±0.25 a	0.64±0.04 b	0.13±0.01 b
	Feces	0.02±0.00 b	0.03±0.00 b	0.31±0.02 a	0.25±0.02 a
Phenylalanine (Phe)	Body	27.55±1.07 a	28.64±1.13 a	19.20±1.23 b	15.73±1.19 b
	Feces	11.56±0.81 b	9.62±0.57 b	23.69±1.04 a	18.96±1.07 a
Proline (Pro)	Body	51.07±3.32 a	50.59±4.81 a	59.13±5.57 a	56.36±2.97 a
	Feces	21.83±1.54 a	24.16±1.47 a	32.77±2.05 a	40.54±3.22 a
Serine (Ser)	Body	55.50±2.11 a	55.86±4.08 a	41.88±2.58 b	34.26±2.14 b
	Feces	13.27±1.01 b	11.67±0.72 b	31.84±1.22 a	29.62±1.57 a
Threonine (Thr)	Body	37.91±3.84 a	38.90±2.26 a	34.98±3.11 a	37.65±1.89 a
	Feces	24.55±1.86 a	22.88±1.64 a	27.49±2.27 a	30.51±2.72 a
Tyrosine (Tyr)	Body	26.02±1.47 a	24.73±1.61 a	23.45±2.12 a	24.45±2.00 a
	Feces	7.71±0.59 a	6.90±0.47 a	8.93±0.88 a	12.31±0.75 a
Valine (Val)	Body	67.76±6.44 a	68.30±6.13 a	60.67±3.97 a	54.26±4.01 a
	Feces	15.42±1.09 a	18.99±1.45 a	24.01±1.88 a	26.49±2.09 a
Total	Body	814.74±46.77 a	831.04±53.23 a	714.51±37.49 a	716.21±38.91 a
	Feces	295.08±19.44 a	317.10±20.33 a	458.24±34.17 a	498.68±24.53 a

Free amino acid contents are analyzed with a Beckman 6300 Amino Acid Analyzer. Difference of the content of each amino acid among treatments is analyzed by ANOVA followed by the Tukey-Kramer test. Data that do not share the same lowercase or uppercase letter are significantly different at P values of 0.05 or 0.01.
